# Diagnostic Value of Microscopy, Galactomannan, and PCR in *Aspergillus* Culture‐Positive BALF Samples: A Laboratory‐Based Pilot Study

**DOI:** 10.1111/myc.70103

**Published:** 2025-08-06

**Authors:** Miriam Govrins, Roya Vahedi‐Shahandashti, Cornelia Lass‐Flörl

**Affiliations:** ^1^ Institute of Hygiene and Medical Microbiology Medical University of Innsbruck Innsbruck Austria

**Keywords:** airway‐colonisation, *Aspergillus*‐specific PCR, bronchoalveolar lavage (BALF), culture‐positivity, direct microscopy, galactomannan, invasive aspergillosis diagnosis

## Abstract

**Background:**

Diagnosing invasive aspergillosis (IA) remains challenging despite the availability of various tests due to the limited sensitivity and variability in accuracy depending on the clinical context. Laboratory‐based definitions consider different mycological criteria, such as culture and galactomannan (GM) positivity, equivalent in diagnostic weight. However, a more detailed analysis is essential for reliably distinguishing true infection from colonisation.

**Objectives:**

This laboratory‐based pilot study aimed to evaluate the diagnostic reliability of culture positivity by comparing it with fungal microscopy, GM testing, and *Aspergillus*‐specific PCR in bronchoalveolar lavage fluid (BALF) samples, all of which were culture‐positive for *Aspergillus*.

**Materials and Methods:**

Ninety‐two *Aspergillus fumigatus* culture‐positive BALF specimens were obtained from mixed patient populations, displaying various risk factors for IA. The multi‐assay approach used direct microscopy, GM, and *Aspergillus*‐specific PCR. The diagnostic value of each test was assessed utilising a composite score based on mycological findings and clinical suspicion.

**Results:**

Among 92 culture‐positive BALF samples, positivity rates for microscopy, GM, and PCR were 12.0% (*n* = 11), 27.2% (*n* = 25), and 28.3% (*n* = 26), respectively. Notably, in 58.7% (*n* = 54) of cases, culture positivity was not supported by any other mycological test. Direct microscopy showed the strongest correlation with other diagnostic methods, whereas GM and PCR showed moderate agreement.

**Conclusions:**

Based on our data, the current practice of weighing all mycological parameters equally should be reconsidered, with greater emphasis on microscopy and multimodal diagnostics rather than on culture alone, particularly in non‐neutropenic patients.

## 1 Introduction

*Aspergillus fumigatus* is the predominant species involved in aspergillosis and has been designated a critical priority fungal pathogen by the World Health Organisation [1]; as its spores are primarily airborne, the respiratory tract serves as the main site of infection, particularly in individuals with underlying sinus or pulmonary conditions [2, 3]. While conidia facilitate exposure and initial colonisation, germination from conidia to hyphae and hyphal propagation are the key pathogenic steps in the development of invasive pulmonary aspergillosis [3, 4].

Various guidelines for the standardised diagnosis of invasive fungal disease have been published, including ESCMID‐ECMM‐ERS [5], EORTC/MSGERC criteria [6], and specific guidelines for IAPA [7], CAPA [8], and ICU patients [9, 10]. Common mycological criteria across these guidelines, which are often considered equivalent for the diagnosing of aspergillosis, include microscopic detection of mould elements in BALF, positive cultures, positive PCR, and a galactomannan antigen index > 0.5 in plasma/serum and/or > 0.8 [6, 9] or > 1.0 in BALF [8, 10]. Although the armamentarium of diagnostic methods has broadened [11], early, sensitive, and specific diagnosis of *Aspergillus*‐caused infections remains challenging, and the distinction between its colonisation and invasion of the respiratory tract is also unclear. Isolating *Aspergillus* spp. from respiratory tract specimens, such as BALF, by culture may have low sensitivity and does not always indicate infection, as colonisation is common, especially in asymptomatic patients without invasive or allergic disease [12, 13]. Furthermore, culture‐proven *Aspergillus* colonisation is found in up to 30% of patients with chronic obstructive pulmonary disease (COPD) [14]. Hence, a positive *Aspergillus* culture from BALF may not always be reliable for diagnosis, either as a sole diagnostic criterion or when considered equivalent to other diagnostic methods. Therefore, distinguishing invasive disease from colonisation is critical to avoid misdiagnosis and unnecessary antifungal therapy. This distinction often requires additional tests that reflect the invasiveness of *Aspergillus* infection, associated more with the hyphae than with conidia, such as microscopy or GM testing, which are typically positive only when fungal hyphae are present [15, 16]. The present laboratory‐based pilot study aimed to analyse the BALF culture‐positive samples using microscopy, *Aspergillus* PCR, and GM testing to evaluate whether all diagnostic criteria should truthfully be regarded as interchangeable in at‐risk, mainly non‐neutropenic patients, including critically ill ICU patients, those with chronic lung disease, solid organ transplant recipients, individuals on immunosuppressive therapy (e.g., corticosteroids or biologics), and patients with diabetes, malnutrition, or indwelling medical devices.

## 2 Material and Methods

### 2.1 Routine and Extended Mycological Analysis of Fungal Cultures

A total of 92 *A. fumigatus* positive BALF samples collected from the routine laboratory at the Institute of Hygiene and Medical Microbiology, Medical University of Innsbruck were selected for further analysis. Standard laboratory procedures include fungal culture, and additional tests are performed only when specifically requested by the physicians. All samples were obtained from mixed non‐neutropenic patients, meeting criteria such as prolonged or high‐dose corticosteroid use, severe influenza, organ transplants, COPD with acute exacerbation, and mechanical ventilation, displaying a low to intermediate risk for IA. Samples were centrifuged at 2000 rpm for 10 min, and the sediment was inoculated onto Sabouraud chloramphenicol 2 agar (bioMérieux, Vienna, Austria) and Oatmeal agar (Condalab, Madrid, Spain) using a swab. Plates were incubated at 28°C for 7 days. MALDI‐TOF MS (Matrix‐Assisted Laser Desorption/Ionisation Time‐of‐Flight; Bruker Daltonik, Bremen, Germany) using the Biotyper library v4.1 [17] was applied for species identification. Following routine diagnostics, the leftover material from 92 *A. fumigatus* culture‐positive BALF samples underwent comprehensive evaluation, including fungal microscopy, GM testing, and *Aspergillus*‐specific PCR.

Microscopy was performed via calcofluor white staining (Fungi‐FluorTM, Polyscience Inc., Warrington, PA, USA). GM testing was performed using the IMMY Sona *Aspergillus* GM Lateral Flow Assay (IMMY Diagnostics, Oklahoma, USA), following the manufacturer's instructions. A threshold of 0.5 was used to define positivity, and results were read with the Sona LFA Cube Reader (IMMY Diagnostics, Oklahoma, USA). DNA was extracted from centrifuged BALF samples using the SepsiTest‐UMD Kit (Molzym GmbH, Bremen, Germany), according to the manufacturer's protocol. *Aspergillus* PCR testing was performed using the MycoReal Kit *Aspergillus* (Ingenetix, Vienna, Austria), following the manufacturer's instructions. *A. fumigatus* PCR was defined as positive with a cycle threshold < 38.0.

### 2.2 Spiking Experiment: Effect of *Aspergillus* Spores on GM and PCR Testing

To simulate the early interaction of inhaled *A. fumigatus* conidia with the airway environment, representing the initial phase that may precede colonisation, a spiking experiment was performed to assess their impact on GM and *Aspergillus*‐specific PCR test results. *Aspergillus* culture‐negative BALF samples were spiked with *A. fumigatus* spores at concentrations of 100, 1000, and 10,000 spores/mL, at levels comparable to daily inhalation exposure [18, 19]. GM and *Aspergillus*‐specific PCR testing were performed under two conditions: first, immediately after spiking negative BALF samples with *A. fumigatus* conidia; and second, following overnight incubation at 37°C to assess the impact of the formation of hyphae on test results.

### 2.3 Composite Score Calculation

A composite score was developed to estimate the likelihood of IA by summing positive findings from mycological tests and clinical suspicion. One point was assigned for each of the following: positive fungal culture, microscopy, GM, *Aspergillus*‐specific PCR, additional samples within 2 weeks showing positive mycological results, and clinical suspicion (Table 1). Clinical suspicion was defined as two or more mycological tests requested by the clinician (culture, microscopy, GM, and *Aspergillus* PCR), noting that fungal culture is routinely performed on all BALF samples irrespective of request. Its inclusion in the composite score was designed to reflect the diagnostic reasoning and context given to clinical microbiology laboratories. Higher composite scores were interpreted as higher probability of IA. The proposed score is designed to provide a structural approach for synthesising multiple test results into a single, interpretable value to aid in diagnosis and assessment of IA.

**TABLE 1:** Composition of diagnostic score. The score was calculated by summing positive mycological test results and clinical suspicion of invasive aspergillosis (IA), with higher scores indicating a greater likelihood of IA.

Category	Parameters	Points
Mycological parameters	Positive *Aspergillus* culture	1
	Positive fungal microscopy	1
	Positive BALF GM	1
	Positive *Aspergillus* PCR	1
Clinical suspicion	Two or more specific mycological tests requested	1
Additional criteria	Additional sample/s within 2 weeks with positive mycological parameters	1
Maximal number of points		6

Abbreviation: GM, galactomannan.

### 2.4 Data Management and Statistical Analysis

All data were anonymized. Statistical analysis was performed using SPSS software (Version 29.0; IBM Corp., Armonk, NY), and graphics were created using BioRender.com.

## 3 Results

**FIGURE 1:**
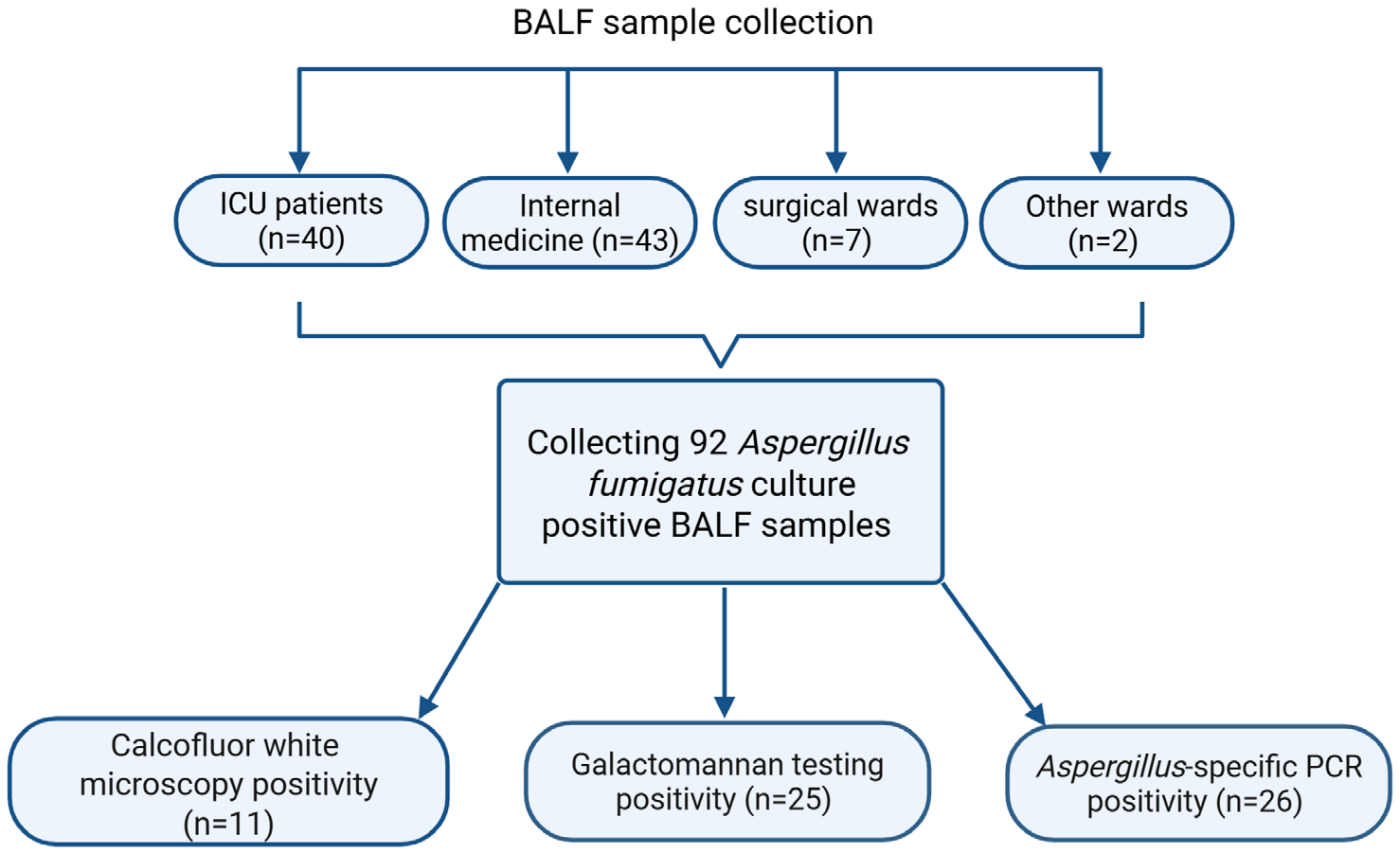
Schematic representation of the bronchoalveolar lavage fluid (BALF) sample collection and testing workflow, including the proportion of positive results for each test.

A total of 92 *A. fumigatus* culture positive BALF samples from individual patients were analysed. Of those, 43.5% (*n* = 40) were from patients admitted to an ICU at the time of sampling. The remaining samples came from internal medicine (*n* = 43; 46.7%), surgical wards (*n* = 7; 7.6%), and other wards (*n* = 2; 2.2%). Ten patients were admitted to transplantation units (ICU and non‐ICU; 10.9%). Sixty‐two percent of the patients were male (*n* = 57), 38% female (*n* = 35). All of the samples underwent calcofluor white microscopy, GM testing, and *Aspergillus*‐specific PCR, with positivity rates of 12.0% (*n* = 11), 27.2% (*n* = 25) and 28.3% (*n* = 26), respectively. Figure 1 provides a schematic overview of the collected sample data and the testing results.

**FIGURE 2:**
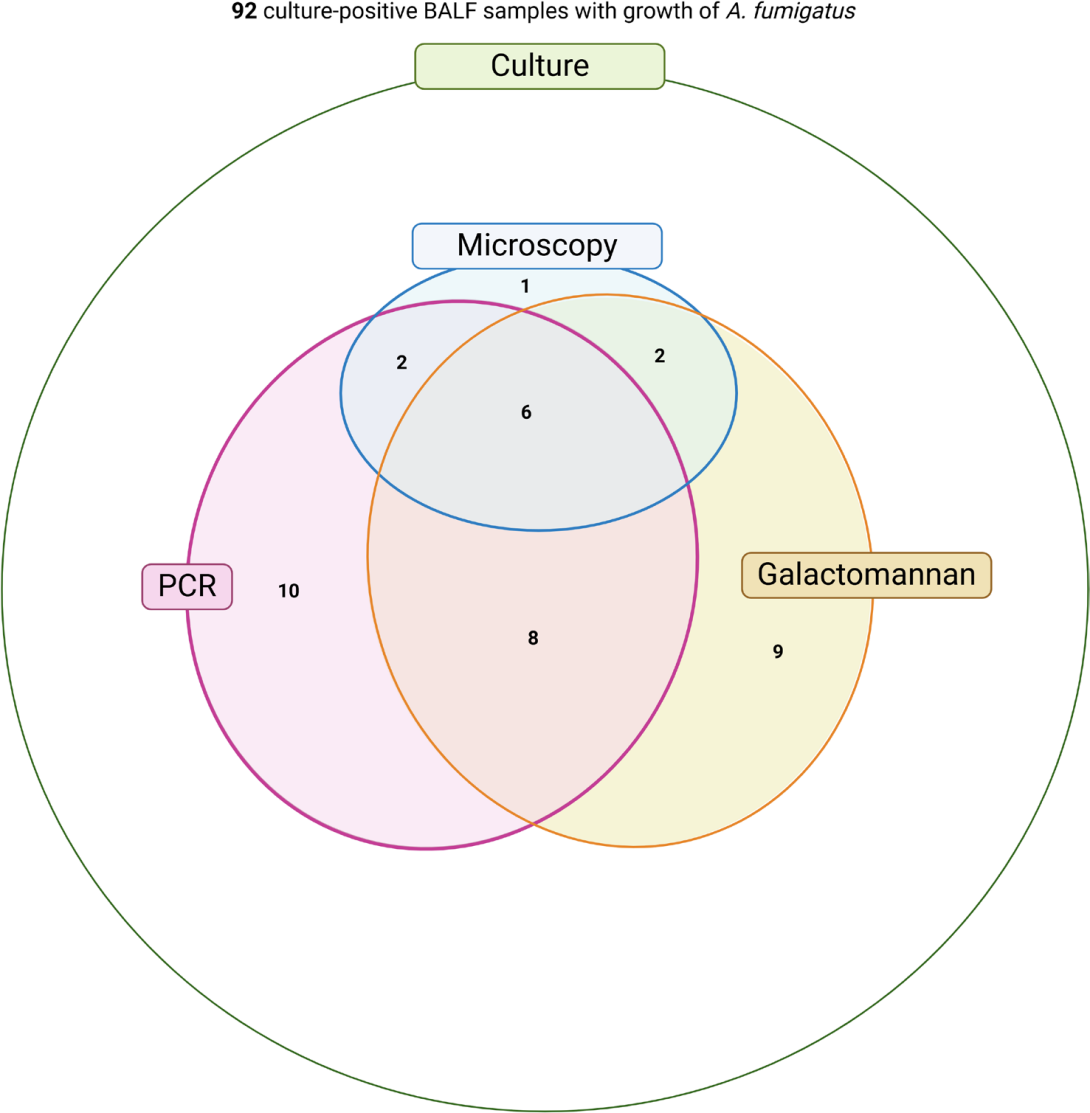
Venn diagram illustrating the overlap of positive results from direct microscopy, *Aspergillus* PCR, and galactomannan testing in 92 *A. fumigatus* culture‐positive bronchoalveolar lavage fluid (BALF) Samples.

Among the culture‐positive BALF samples, six showed concordant positivity across all three tests: microscopy, GM, and PCR. Of the eleven microscopy‐positive samples, ten were also positive in at least one of the other two tests (GM or PCR), while GM and PCR each had additional cases with positivity. The overlap between test results is visualised in the Venn diagram shown in Figure 2. Notably, in 58.7% of cases (*n* = 54), culture was the only positive mycological criterion, with no confirmation by any of the other diagnostic methods (Figure 2).

A composite score representing the sum of positive mycological test results (culture, microscopy, GM, PCR, additional criteria; Table 1) and clinical suspicion of IA was calculated where each positive feature was counted as one point. Clinical suspicion was the case for 20.7% of patients (*n* = 19). Composite scores of ≥ 3 were considered high and suggestive of invasive fungal disease. These scores were observed in 28.3% (≥ 3; *n* = 26) of cases, and 16.3% (≥ 4; *n* = 15), respectively.

**FIGURE 3:**
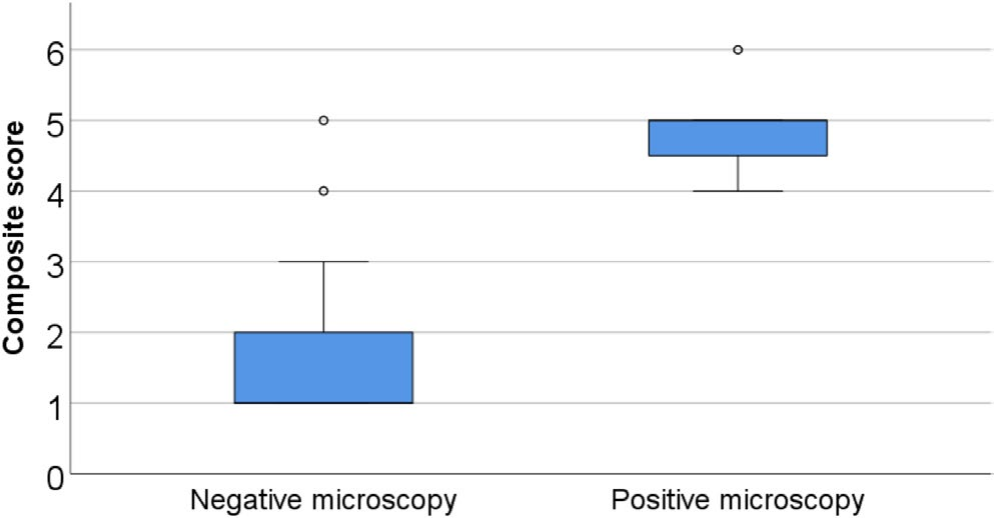
Boxplot of composite scores by direct microscopy result. The clinical score represents the sum of mycological test results and clinical suspicion of invasive aspergillosis; as detailed in Table 1. Patients with positive microscopy (septate hyphae detected, *n* = 11) exhibited significantly higher composite scores (median = 5, IQR = 1) compared to those with negative microscopy (no septate hyphae detected, *n* = 81; median = 1, IQR = 1). Individual outliers are indicated as dots.

The Pearson Chi‐Square test revealed a very strong and statistically significant association between microscopy positivity and a diagnostic score ≥ 4 points (*Φ* = 0.84, *p* < 0.001). This finding was further supported by a point‐biserial correlation (Pearson), which demonstrated a strong and statistically significant association between high diagnostic scores and microscopy positivity (*r* = 0.744, *p* < 0.001). Additionally, comparison of composite scores between microscopy‐negative and microscopy‐positive samples revealed that patients with positive direct fungal microscopy (septate hyphae detected, *n* = 11) had significantly higher composite scores (median = 5, IQR = 1) than those with negative microscopy (no septate hyphae detected, *n* = 81; median = 1, IQR = 1) (Figure 3). GM (*Φ* = 0.51, *p* < 0.001) and PCR alone (*Φ* = 0.47, *p* < 0.001) were only moderately associated with scores ≥ 4 points, while their combination showed a stronger association with scores ≥ 4 points (*Φ* = 0.58, *p* < 0.001). In the subset of 22 GM‐positive cases, the mean GM index (GMI) value was 9.06 (SD = 8.43), with values ranging from 0.56 to 24.72. A Spearman's rho correlation analysis revealed a statistically significant correlation between the composite score and the GMI (*ρ* = 0.64, *p* < 0.001). The Pearson correlation analysis between GMI and microscopy positivity revealed a significant correlation (*r* = 0.55, *p* < 0.001), indicating an association between higher GMI values and higher scores as well as microscopy positivity, respectively. Among GM‐positive samples, those with microscopy positivity had significantly higher GMI values (median 15.4 vs. 3.2; *p* < 0.001, Mann–Whitney *U* test). In summary, the strong association between high composite scores (≥ 4 points) and microscopy positivity (*Φ* = 0.84, *p* < 0.001) (Figure 3) suggests that microscopy positivity is a highly specific indicator of probable IA, while GM positivity and *Aspergillus* PCR alone are less specific. The combination of the latter two tests and high GMI values were associated with a higher likelihood of IA, especially when corroborated by microscopy positivity.

The results of GM testing and *Aspergillus* PCR on fungal culture‐negative BALF samples spiked with *A. fumigatus* spores are displayed in Table 2. GM testing was negative when the samples were tested immediately after being spiked (non‐incubated), but turned positive after overnight incubation, suggesting that GM detection depends on fungal growth stage (hyphae formation). PCR testing was positive in all spiked samples except for the lowest concentration of spores in the non‐incubated group. In that case, the cycle threshold (Ct) value exceeded the positivity cut‐off, so the result was considered negative.

**TABLE 2:** Performance of galactomannan (GM) and *Aspergillus* PCR in bronchoalveolar lavage fluid (BALF) spiked with different concentrations of *Aspergillus fumigatus* spores before and after incubation.

Initial spore concentration per mL		GM		*Aspergillus* PCR	
		Result	GMI	Result	Cycle threshold
Direct testing	100	Negative	0.09	Negative	40.39
	1000	Negative	0.14	Positive	34.48
	10,000	Negative	0.13	Positive	32.79
After incubation	100	Positive	0.71	Positive	33.32
	1000	Positive	15.79	Positive	27.07
	10,000	Positive	23.58	Positive	22.59

*Note:* The table shows the diagnostic results for Galactomannan Index (GMI) and PCR (cycle threshold values) at varying initial spore concentrations (100, 1000, and 10,000 spores/mL).

## 4 Discussion

Mycological culture remains a fundamental and widely available method for detecting fungal infections [20, 21]; however, its diagnostic value is limited when used as a standalone tool or regarded as equivalent to other diagnostic criteria. A positive BALF culture, even in patients at medium risk for IA, raises questions about the need for antifungal therapy, highlighting the necessity to further analyse BALF culture positivity. Equating culture positivity with other mycological criteria when diagnosing infection can lead to diagnostic uncertainty and potentially unnecessary antifungal therapy, highlighting the need for cautious interpretation of fungal culture results from respiratory specimens [22]. The present study aimed to evaluate the diagnostic relevance of *Aspergillus* positive‐culture BALFs in comparison with other mycological criteria, including fungal microscopy, GM, and *Aspergillus*‐specific PCR, and to provide evidence‐based guidance for their optimal interpretation and use in clinical practice.

Our findings showed that culture positivity alone had limited diagnostic value, as over half (58.7%) of culture‐positive samples were not supported by other mycological criteria (Figure 2). This finding reinforces previous criticisms regarding culture positivity as a reliable diagnostic method [23]. A prior study reported that only 1.6% of 121 patients with positive *Aspergillus* cultures met the EORTC‐MSG criteria, with the rest considered likely incidental findings, possibly due to contamination through inhaled spores in sputum samples [23]. This is particularly relevant in ICU patients (*n* = 40; 43.5% of our study population), where the absence of classical host factors, combined with potential environmental exposure prior to hospital admission, further complicates the interpretation of mycological findings [10]. Therefore, positive *Aspergillus* cultures should be interpreted in light of both clinical and microbiological contexts, as culture positivity may result not only from IA but also from colonisation. In the current guidelines, culture positivity in a fitting clinical context automatically meets the mycological criteria. Given the potential for false‐positive *Aspergillus* cultures due to colonisation, and in light of our finding that many culture‐positive cases lacked other supporting mycological criteria, we suggest multimodal testing, especially in culture‐positive samples, to allow a more informed interpretation of positive results. From a laboratory perspective, further investigation of positive *Aspergillus* cultures may lead to more reliable diagnoses of IA, as their diagnostic value appears to be lower compared to microscopy, GM, and PCR testing.

Our findings strongly support the diagnostic value of direct microscopy in BALF samples for the rapid confirmation of IA in preselected patients. Microscopy positivity was significantly associated with a higher composite diagnostic score (≥ 4 points) (Figure 3), indicating probable IA in patients with a corresponding clinical presentation. All microscopy‐positive samples, except for one, were also positive in GM testing and/or *Aspergillus* PCR (Figure 2), further supporting the concordance of microscopic findings with serological and molecular‐based methods. Discrepancies observed in the Venn diagram, such as microscopy‐positive but GM‐/PCR‐negative results, or vice versa, reflect the importance of multimodal testing. GM positivity with negative microscopy was often associated with low GMI, suggesting a lower fungal burden. Conversely, positive microscopy and negative GM and/or PCR results could be caused by an irregular distribution of fungal material within the sample, low antigen and DNA concentrations, or sample degradation affecting molecular and antigenic targets but not hyphal structures. Recently, the Indian guideline for COVID‐19‐associated pulmonary mucormycosis emphasised the importance of direct microscopy, highlighting its higher reliability compared to culture, particularly in cases lacking strong clinical or radiological suspicion [24]. In contrast, current guidelines for IA continue to weigh all mycological criteria equally [5, 6, 7, 8, 9, 10]. However, our findings suggest that despite its lower sensitivity [25], direct microscopy should be prioritised in suspected cases of IA, as the visualisation of septate hyphae provides a rapid and highly suggestive result [16].

GM testing in BALF samples has demonstrated strong diagnostic utility in various studies, particularly when used in combination with other diagnostic criteria [10, 26]. GM testing has been widely validated as a valuable tool, with previous studies reporting high sensitivity (74%–92%) and specificity (81%–91%) in culture‐positive BALF samples [15]. Our data corroborate these findings, as we observed a moderate to strong association between GM positivity and the composite diagnostic score. Interestingly, GM remained negative in BALF samples spiked with *Aspergillus* spores but turned positive after incubation, confirming that the test detects fungal hyphae rather than the mere presence of spores (Table 2). While false positives due to cross‐reactivity with other moulds can be ruled out in our patients, it remains technically possible that mould‐active prophylaxis contributed to false‐negative results [15, 27].

Although highly sensitive, PCR testing showed certain limitations, particularly in its susceptibility to detecting the presence of spores. In spiked BALF experiments, PCR yielded positive results even at low spore concentrations, suggesting that spores alone, without active infection, can lead to PCR positivity. This highlights the importance of interpreting PCR results within the appropriate clinical context and considering pre‐test probability, as previous studies have demonstrated that the predictive value of PCR can vary significantly depending on the patient population [28, 29]. PCR can provide excellent diagnostic accuracy, particularly when used in combination with GM. Studies have reported up to 100% sensitivity and 98% specificity when both tests are positive [28]. This combined approach enhances diagnostic reliability, as also reflected in our data, where concurrent positivity of PCR and GM was strongly associated with a higher likelihood of IA. There are some limitations in the present study that should be considered. Firstly, the composite diagnostic score used has not been previously validated and serves as a proxy for the information, warranting further investigation for validation. Additionally, the lack of detailed clinical data, such as patient symptoms, risk factors, and radiological findings, is a key limitation, preventing the determination of the rate of proven or probable IA cases in our cohort. Clinical suspicion was documented in only 20.7% of cases, highlighting the variability in pre‐test probability, which significantly influences the diagnostic performance of PCR, GM, and other mycological tests depending on the clinical context [10, 15, 30].

In conclusion, our findings highlight the importance of a multimodal diagnostic approach, particularly GM, PCR, and microscopy, for the diagnosis of IA. The practice of weighing all mycological parameters equally should be reconsidered, with greater emphasis placed on microscopy and multimodal testing rather than relying on fungal culture alone, depending on the laboratory's equipment.

## Author Contributions

**Miriam Govrins:** conceptualization, methodology, writing – review and editing, writing – original draft, formal analysis, investigation, validation, visualization, data curation, software, project administration. **Roya Vahedi‐Shahandashti:** conceptualization, methodology, writing – original draft, writing – review and editing, visualization. **Cornelia Lass‐Flörl:** conceptualization, methodology, writing – original draft, writing – review and editing, funding acquisition, supervision, resources.

## Ethics Statement

BALF samples for this study were leftovers of routinely processed samples and collected with approval of the ethics committee of the Innsbruck Medical University (Ethic Number: UN4926/2013).

## Consent

As the samples were anonymised and only non‐identifiable information (sex and medical ward) was collected, no personally identifiable data were included, and written informed consent from patients was not required.

## Conflicts of Interest

The authors declare no conflicts of interest.

## Data Availability

All data supporting the findings of this study are included in the article. Additional raw data are available from the corresponding authors upon request.
